# The correlation between the expression of differentiated embryo-chondrocyte expressed gene l and oral squamous cell carcinoma

**DOI:** 10.1186/2047-783X-19-21

**Published:** 2014-04-23

**Authors:** Jingmin You, Lu Lin, Qiong Liu, Tengfei Zhu, Kun Xia, Tong Su

**Affiliations:** 1 Department of Oral and Maxillofacial Surgery, Xiangya Hospital, Central-South University, Changsha 410008, China; 2 Department of Stomatology, First People’s Hospital of Changde, Changde 415003 Hunan, China; 3 State Key Laboratory of Medical Genetics, Central South University, 110 Xiangya Road, Changsha, Hunan 410078, China

**Keywords:** Differentiated embryo-chondrocyte expressed gene l, Immunohistochemistry, Metastasis, Oral squamous cell carcinoma, Real-time polymerase chain reaction, Recurrence

## Abstract

**Background:**

This study aims to explore the correlation between expression of differentiated embryo-chondrocyte expressed gene l (DEC1) and oral squamous cell carcinoma (OSCC), which could provide the reference for treatment and prognosis assessment of OSCC.

**Methods:**

The expression of DEC1 in tissues from 56 primary OSCC patients and 20 normal oral mucosa samples were detected using real-time polymerase chain reaction and immunohistochemical methods, respectively.

**Results:**

The results showed that the positive expression rate of DEC1 in the OSCC group was significantly higher than that in the normal group (*P* <0.05); further, the expression of DEC1 in different OSCC groups was statistically significant (*P* <0.05). The expression of DEC1 in the 1-year recurrence OSCC group was significantly higher than other groups. The expression of DEC1 in the 3-years no recurrence OSCC group was the lowest.

**Conclusions:**

The expression of DEC1 was associated with the incidence of OSCC and there was a negative correlation between the expression of DEC1 and the prognosis of OSCC.

## Background

Oral squamous cell carcinoma (OSCC) is the most common malignant tumor in the oral and maxillofacial region; lymph node metastasis occurs at an early stage and distant metastasis through the blood circulation is advanced. Despite operation plus radiotherapy, chemotherapy, and other comprehensive sequence therapies, the 5-year survival rate for OSCC patients has not improved significantly. In particular, recurrence or metastasis of cervical lymph nodes and distant metastasis shortly after surgery frequently occur. Therefore, it is imminent to pay more attention to the study of tumor recurrence and metastasis genes for OSCC.

A previous study [1] has confirmed the over-expression of the differentiated embryo-chondrocyte expressed gene l (DEC1) gene in OSCCs. This study will focus on the correlation between prognosis of OSCC and expression of DEC1, which could provide guidance for evaluating prognosis and improve the treatment of OSCC.

## Methods

### Ethical statement

Any experimental research that is reported in the manuscript has been performed with the approval of the Research Ethics Committee of Xiangya Hospital of Central South University. Research carried out on humans was in compliance with the Helsinki Declaration.

### Patients and specimen collection

A total of 56 patients (39 male, 17 female; age, 37–62 years old) who were diagnosed with OSCC (high differentiation, grade III to IV, preoperative without radiotherapy and chemotherapy) by pathological diagnosis in the Department of Oral and Maxillofacial Surgery of Xiangya Hospital of Central South University from January 2008 to October 2012, were recruited. The tumor samples were divided into two types. One group of samples was immediately frozen in liquid nitrogen and stored at −80°C; the remaining samples were embedded in paraffin for immunohistochemistry (IHC). According to the criteria of UICC TNM staging classification system for oral and maxillofacial tumor in 2002 [2], they were all high differentiated carcinoma. TNM stage: 35 cases in stage III, 21 cases in stage IV; 51 cases had lymph node metastasis; 42 cases had tissue metastasis and recurrence within 3 years (14 cases had tissue metastasis and recurrence within 1 year, 14 cases had tissue metastasis and recurrence in within 1 to 2 years, and 14 cases had tissue metastasis and recurrence within 2 to 3 years), 14 cases had no tissue metastasis or recurrence in 3 years. At the same time, 20 cases of normal oral mucosa were randomly divided into 4 groups as a control.

### Real-time quantitative polymerase chain reaction

The total RNA was extracted with the TRIzol method. They were performed according to the manual of tissue RNA extraction TRIzol Reagent kit (Invitrogen, CA, USA). Briefly, 2 μg total RNA was taken from each sample to reverse transcription to cDNA according to the manual of RevertAid First StrandcDNA Synthesis Kit (Thermo Scientific, Pittsburgh, PA, USA), and then PCR was performed with SYBR® Premix Ex Taq kit (TaKaRa Biotechnology Co., Ltd, Dalian, China). The primers for DEC1 were as follows: F: 5'-TTTTACTGATGCCCTGCACA-3'; R: 5'-CAATCTGCAATTCCCTCTGC-3'. The primers for β-actin, which was used as an internal reference, were as follows: F: 5'-GGCATGGGTCAGAAGGATT-3'; R: 5'-TGGTGCCAGATTTTCTCCA-3'. Reaction conditions were as follows: 95°C for 30 s, 1 cycle; 95°C for 5 s and 60°C for 30 s, 40 cycles. The 2^–ΔΔCt^ method [3] was used to calculate the expression of DEC1 gene in OSCC tissues and normal oral mucosa tissues.

### Immunohistochemistry detection

Pathological paraffin blocks were cut into 4 μm continuous sections for IHC staining. Following deparaffinization, dehydration, and antigen retrieval, the sections were incubated with 1:200 diluted DEC1 rat monoclonal antibody against human at 4°C overnight. After that, they were washed with PBS and incubated at 37°C in a water bath for 2 h after drop-adding the second antibody and washing with PBS. After treatment with the DAB solution, they were flushed completely, counterstained with hematoxylin, washed with water, treated with dehydration and transparency media, mounted on slides, and observed under the microscope. The IHC staining results were scored by the pathologist and author separately so as to minimize the subjective factors, compared, and the final comprehensive results were obtained. The results were expressed as the staining index score which was calculated as the product of A and B. A was the categorical score of the proportion of positively stained cells in five random microscopic fields: positive cells ≤5% = 0; 6% to 25% positive cells = l; 26% to 50% positive cells = 2; 51% to 75% positive cells = 3; and positive cells >75% = 4. B was the categorical score of the staining intensity: 0 = no positive staining; 1 = light yellow staining; 2 = pale brown staining; 3 = bright brown staining. Each section was observed under the × 400 microscope, for 5 continuous high power fields and 100 cells were observed, taking their mean value. Integration optic density (IOD) values of positive staining screenshots taken by Leica DM5000B micrograph analysis system (Leica DMI5000B, Germany) and calculated using the Image Pro Plus 6.0 Image analysis software. IOD values and positive expression intensity were positively correlated.

### Statistical analysis

All statistical analysis was done using SPSS 18.0 for windows (SPSS, Inc., Chicago, IL, USA). A student two-tailed non-paired *t*-test was used to determine significant differences between treatment and control. *P* <0.05 was considered as statistically significant.

## Results

### Analysis of DEC1 mRNA expression in different prognostic groups of OSCC tissue and normal mucosa tissue

The expression of DEC1 mRNA in different prognosis OSCC groups was significantly higher than that of the normal group; the difference was statistically significant (*P* <0.05, Table 1). The expression of DEC1 mRNA in the 1-year recurrence OSCC group was significantly higher than in the other groups (*P* <0.05). The 3 years without recurrence OSCC group had the lowest expression of DEC1 mRNA among them. The expression of DEC1 mRNA in the normal group was significantly lower than that in the OSCC group (*P* <0.05, Table 1).

**Table 1:** Comparison results of expression of DEC1 gene in different groups

**Group n**		**DEC1**		***t***	***P***
		**Ca (2**^**-ΔΔCt **^**value)**	**Nm (2**^**-ΔΔCt **^**value)**		
1	14	9.73 ± 2.09	1.02 ± 0.18	11.731	0.000
2	14	6.64 ± 0.92^a^	1.05 ± 0.15	16.962	0.000
3	14	5.47 ± 1.15^a^	0.98 ± 0.14	11.226	0.000
4	14	4.31 ± 0.85^ab^	0.94 ± 0.21	10.925	0.000

Group 1: 1-year recurrence OSCC group; Group 2: 1- to 2-year recurrence OSCC group; Group 3: 2- to 3-year recurrence OSCC group; Group 4: 3 years without recurrence OSCC group.

### Expression of DEC1 protein in different prognosis of OSCC

**Figure 1:**
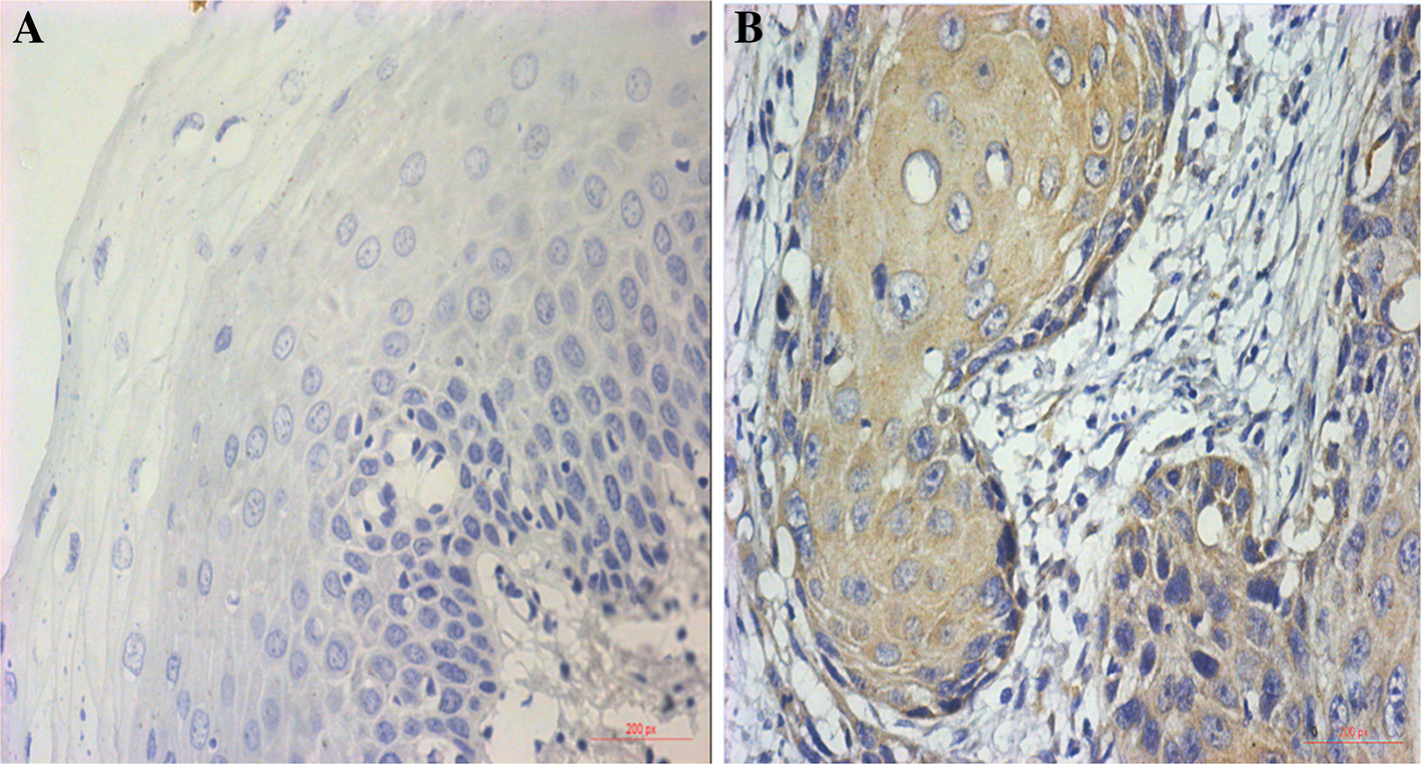
**Expression of DEC1 protein in human normal oral mucosa tissue and OSCC tissue (SP × 400). (A)** Normal oral mucosa tissue (negative), **(B)** OSCC tissue (positive).

**Figure 2:**
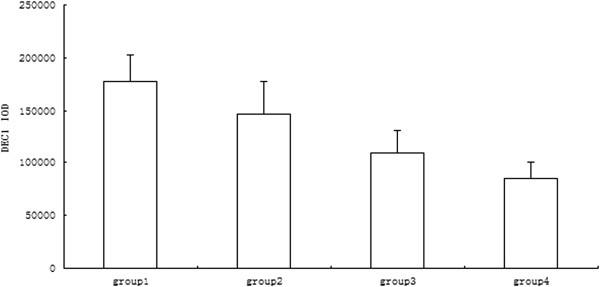
**IOD values of DEC1 in different prognostic OSCC groups.** Group 1: 1-year recurrence OSCC group; Group 2: 1 to 2 years recurrence OSCC group; Group 3: 2 to 3 years recurrence OSCC group; Group 4: 3 years without recurrence OSCC group.

IHC staining results are shown in Figure 1. The staining index score of OSCC tissues was higher than that of normal mucosa tissues, so the expression level of DEC1 in the OSCC group was significantly higher than that of the normal group (*P* <0.05). IOD values of DEC1 expression in different groups are shown in Figure 2. IOD values of DEC1 expression in the 1-year recurrence OSCC group was significantly higher than that of other groups. The 3-year without recurrence group had the lowest IOD value (*P* <0.05).

## Discussion

The DEC1 is a member of the basic helix loop protein family containing the basic helix loop (b HLH) structure. It plays an important role in cartilage formation, growth of axons, biological clock regulation, cell differentiation, and tumor development. The human DEC1 gene is located at p25.3-26 on chromosome 3. Its size is about 5.7 Kb, including 5 exons and 4 introns [4]. There are many transcription factor binding sites, including CAMP and E-box response element, at its 5'-end [5]. DEC1 protein is composed of 412 amino acids and plays a physiological regulating role through the activation of the phosphorylation sites. DEC1 protein is widely expressed in liver, intestine, and cartilage tissues. Studies have found it to be overexpressed in various tumors, including leukemia [6], lung cancer [7], liver cancer [8], renal cell carcinoma [9], gastric cancer [10], and pancreatic cancer [11]. However, to date, there has been no reports regarding the expression of DEC1 in OSCC.

According to previous studies on DEC1 expression and hypoxia-inducible factor 1 alpha (HIF-1α), a high expression of DEC1 in tumor tissue was induced by hypoxia [9,10]. In the hypoxic environment of tumor cells, the expression of DEC1 was up-regulated, which could inhibit the expression of cellular apoptosis-related factors, such as HIF-1α and p53, and promote the growth of tumor cells. The studies have shown that hypoxia can induce the expression of DEC1 in gastric cancer and pancreatic cancer cell lines [9,10]. Zheng et al. [10] found that the expression of HIF-1α and DEC1 is mild and the expression of DEC1 was higher than that of HIF-1α in a normal environment. Hypoxia could induce the expression of DEC1 mRNA but had no significant effect on the expression of HIF-1α mRNA. The expression of DEC1 mRNA was significantly reduced when inhibiting the expression of HIF-1α.

The tight junction protein 1 (claudin-1) is an important inhibition protein of tumor infiltration function. Liu et al. [12] studied the correlation between expression of claudin-1, DEC1, and invasion of breast cancer. They found that the expression of DEC1 is positively correlated with grade of breast cancer and negatively correlated with the expression of claudin-1. Further experiments found that the expression of claudin-1 mRNA and protein level were enhanced and reduced cell invasive ability after DEC1 gene silencing in breast cancer cell lines (MCF-7 and MDA-MB-231). Therefore, overexpression of DEC1 may be adjusted through the mechanism of down-regulation of claudin-1 to promote the invasion and metastasis of breast cancer.

The classic tumor suppressor p53 gene can be activated under the conditions of hypoxia, DNA damage, and oncogene activation, which mediate the blocking of cell periodic apoptosis and the aging process. A study found that DEC1 and p53 could form a feedback loop to control apoptosis induced by DNA damage through macrophage inhibitory cytokine 1 (MIC-1) [13]. DEC1 inhibited the combination of MIC-1 gene promoter and p53 following DNA damage and reduced the expression of MIC-1. The regulating mechanism between DEC1 and p53 may promote the development of tumors.

Epithelial mesenchymal transition (EMT) is an important step in the invasion and migration of tumor cells. Wu et al. [14] studied the role of DEC1 in EMT *in vitro* with human PANC-1 cells. They found that TGF-β up-regulated the expression of DEC1 and regulated the expression of EMT localization-related factors such as phosphorylated Smad3 (pSmad3), Claudin-4, and N-cadherin in PANC-1 cells. In the presence of TGF-β during DEC1 gene silencing, the EMT process was inhibited and TGF-β induced PANC-1 cells to adopt a fusiform shape. In addition, synergistic effects of the expression of DEC1 and TGF-β were closely related to the invasion and migration of PANC-1 cells. These findings suggest that DEC1 plays an important role in promoting tumor cell invasion and metastasis by regulating EMT-related factors in pancreatic cancer.

## Conclusions

This experiment found that in OSCC patients (stage III to IV), the expression of DEC1 in different prognosis groups had a significant difference (*P* <0.05). The expression of DEC1 in the 1-year recurrence group was significantly higher than in other groups. The expression of DEC1 in the 3 years without recurrence group was the lowest. These data showed that the expression of DEC1 may be negatively correlated to the prognosis of OSCC. The results further defined the important role of DEC1 in promoting tumor invasion and metastasis.

## Abbreviations

DEC1: Differentiated embryo-chondrocyte expressed gene l; EMT: Epithelial mesenchymal transition; HIF-1α: Hypoxia-inducible factor 1 alpha; IHC: Immunohistochemistry; IOD: Integration optic density; MIC-1: Macrophage inhibitory cytokine 1; OSCC: Oral squamous cell carcinoma.

## Competing interests

The authors declare that they have no competing interests.

## Authors’ contributions

JMY and TS designed the study; JMY, KX, and LL carried out the molecular studies; QL and TFZ carried out the immunoassays; JMY, KX, and TS performed the statistical analysis; JMY, LL, and TS were involved in drafting the manuscript. All authors read and approved the final manuscript.
